# Nail-Patella Syndrome in a 76-Year-Old Woman: Multisystem Phenotype, Diagnostic Delay, and Clinical Implications

**DOI:** 10.3390/jcm15207861

**Published:** 2026-10-12

**Authors:** Cosmina Diaconu, Anca Vidrighin, Emi Marinela Preda, Cristinel Gabriel Diaconu, Carmen Daniela Domnariu, Florina Ligia Popa, Adriana Maria Canciu, Oana-Georgiana Cernea, Dana M. Harris, Corina Porr

**Affiliations:** 1 Department of Nursing and Dental Medicine, Faculty of Medicine, Lucian Blaga University of Sibiu, 550024 Sibiu, Romania; cosmina.diaconu@ulbsibiu.ro (C.D.); carmen.domnariu@ulbsibiu.ro (C.D.D.);; 2 Clinic of Physical Medicine and Rehabilitation, County Clinical Emergency Hospital Sibiu, Bulevardul Corneliu Coposu 2–4, 550245 Sibiu, Romania; 3 Department of Paediatrics, Faculty of Medicine, Lucian Blaga University of Sibiu, 550169 Sibiu, Romania; 4 Department of Radiology, Carol Davila University of Medicine and Pharmacy, 050474 Bucharest, Romania; 5 Department of Radiology and Medical Imaging, Foișor Clinical Hospital of Orthopaedics, Traumatology and Osteoarticular TB, 021382 Bucharest, Romania; 6 Department of Physiology, Faculty of Medicine, Lucian Blaga University of Sibiu, 2A Lucian Blaga Street, 550169 Sibiu, Romania; 7 Department of Radiology, Pneumology and Tuberculosis Hospital, 550196 Sibiu, Romania; 8 Department of Physical Medicine and Rehabilitation, Faculty of Medicine, Lucian Blaga University of Sibiu, 550024 Sibiu, Romania; 9 Doctoral School, Faculty of Medicine, Lucian Blaga University of Sibiu, 550169 Sibiu, Romania; oanageorgiana.spatariu@ulbsibiu.ro; 10 Department of Internal Medicine, Mayo Clinic, Jacksonville, FL 32224, USA; 11 Allergology Department, Faculty of Medicine, Lucian Blaga University of Sibiu, 550169 Sibiu, Romania; 12 Allergology Department, County Clinical Emergency Hospital Sibiu, Bulevardul Corneliu Coposu 2–4, 550245 Sibiu, Romania

**Keywords:** nail-patella syndrome, *LMX1B*, iliac horns, diagnostic delay, case report

## Abstract

Nail-patella syndrome (NPS) is a rare autosomal dominant multisystem disorder characterized by a variable combination of nail abnormalities, patellar hypoplasia or dysplasia, elbow involvement, and iliac horns, with possible renal, ophthalmological, neurological, and skeletal manifestations. Recognition may be substantially delayed when individual abnormalities are assessed separately or attributed to age-related and degenerative musculoskeletal disease. We report the case of a 76-year-old woman evaluated for progressive bilateral knee pain, gait impairment, and chronic multisite musculoskeletal symptoms. Congenital thumbnail abnormalities and long-standing restriction of elbow extension had been present since childhood. Suspicion of NPS was first raised at the age of 43 years after bilateral iliac horns were identified radiographically in one of her grandsons. Clinical examination demonstrated bilateral thumbnail hypoplasia with partial absence of the ulnar nail plate, triangular lunulae, reduced dorsal distal interphalangeal creases, bilateral fifth-finger flexion contractures, marked limitation of elbow extension with restricted pronation and supination, and clinically hypoplastic patellae. Bilateral Lester sign and glaucoma were also documented. Radiographic assessment revealed bilateral patellar hypoplasia, lateral displacement of the left patella, bilateral knee osteoarthritis, and characteristic bilateral iliac horns. Additional findings included bilateral hip osteoarthritis, lumbar degenerative changes, pes planus, a bifid configuration of the left fifth metatarsal, osteopenia, and sensory abnormalities. Walking pain was 70 mm on a 100-mm visual analogue scale, and the WOMAC score was 42/96. Following a 10-day rehabilitation program, walking pain decreased from 70 to 50 mm on the visual analogue scale, while the WOMAC total score improved from 42/96 to 37/96, indicating short-term symptomatic and functional improvement. Routine urinalysis showed no proteinuria or hematuria. Molecular testing for a pathogenic *LMX1B* variant was not performed. The diagnosis was established on the basis of the highly characteristic integrated clinical and radiographic phenotype. This case illustrates how NPS may remain incompletely recognized for decades despite congenital manifestations and emphasizes the clinical value of pattern recognition across multiple organ systems. In particular, the coexistence of characteristic nail changes, patellar abnormalities, elbow involvement, and bilateral iliac horns should prompt consideration of NPS and appropriate multidisciplinary surveillance.

## 1. Introduction

Nail-patella syndrome (NPS), also known as hereditary osteo-onychodysplasia, is a rare autosomal dominant multisystem disorder caused predominantly by pathogenic variants in *LMX1B*. Its prevalence has been estimated at approximately 1 in 50,000 individuals, although the true prevalence may be higher because mildly affected individuals can remain undiagnosed [1,2,3,4].

The characteristic phenotype includes nail dysplasia, patellar hypoplasia or aplasia, elbow abnormalities, and bilateral iliac horns [1,5,6,7,8,9,10]. Nail abnormalities are among the earliest and most recognizable manifestations of NPS, while iliac horns represent a highly characteristic radiographic finding [1,5]. Renal, ophthalmological, neurological, and additional skeletal involvement may broaden the clinical spectrum and contribute to substantial phenotypic variability [1,2,3,4,5].

Because the expression of NPS is highly variable, characteristic findings may be recognized separately rather than as components of a single syndrome, potentially delaying diagnosis. Although delayed recognition into adulthood has been described, the frequency of late diagnosis has not been systematically quantified [1,3,5].

We report the case of a 76-year-old woman with congenital nail abnormalities, bilateral patellar hypoplasia, restricted elbow mobility, bilateral iliac horns, glaucoma, sensory abnormalities, progressive degenerative joint disease, and functional impairment. This case illustrates the diagnostic value of integrating characteristic clinical and radiographic findings and highlights the importance of recognizing the multisystem phenotype of nail-patella syndrome to guide appropriate surveillance and family assessment.

## 2. Case Presentation

This case report was prepared in accordance with the CARE (CAse REport) reporting guidelines to promote transparent and comprehensive reporting of the patient’s clinical history, diagnostic assessment, therapeutic interventions, and follow-up [11]. The completed CARE 2013 reporting checklist is provided as Supplementary Table S1.

### 2.1. Clinical History

A 76-year-old woman presented to the Medical Rehabilitation Department in Sibiu, Romania, with chronic mechanical pain and progressive functional impairment of the lower limbs, predominantly affecting both knees. She also reported pain involving the hips, lumbosacral spine, and ankles; restricted elbow mobility; paraesthesia of the hands and feet; and increasing difficulty with walking.

Her medical history revealed congenital abnormalities of the thumbnails and long-standing limitation of elbow extension since childhood. Despite these findings, nail-patella syndrome had not initially been recognized. Suspicion of NPS was first raised at the age of 43 years, when bilateral iliac horns were identified on pelvic radiography in one of her grandsons. In the context of the patient’s longstanding nail and musculoskeletal abnormalities, this finding prompted reconsideration of the previously fragmented manifestations as part of a possible hereditary multisystem disorder. At that time, NPS was suspected clinically, but no comprehensive multisystem evaluation or molecular confirmation was performed. The diagnosis therefore remained incompletely characterized, and the patient was not followed within a dedicated multidisciplinary surveillance program. No systematic clinical or genetic evaluation of other family members had been performed, and a formal pedigree was not available.

The patient had been diagnosed with glaucoma in her fifties and had a history of osteopenia. Previous ophthalmological evaluations had also documented bilateral Lester sign. She reported a history of left postaxial foot polydactyly, surgically corrected at the age of 6 years, with residual deformity of the lateral forefoot. Persistent vasomotor symptoms consisting of chronically cold hands and feet were also reported.

Bilateral knee pain had been present for approximately 20–25 years, with insidious onset and progressive worsening. The patient denied significant knee trauma, recurrent instability, clicking, or locking. During the preceding decade, mechanical pain and functional limitation had progressively developed in the lumbosacral spine, hips, and ankles, accompanied by increasing difficulty with walking. Symptoms were aggravated by prolonged standing and improved with rest.

A remote traumatic event involving the left foot had occurred approximately 18 years earlier and resulted in fractures of the distal second and third metatarsals. The patient had previously worked as a tailor and remained functionally independent for many years. However, the long-standing elbow abnormalities had caused difficulty during occupational activities, illustrating the cumulative functional impact of the musculoskeletal phenotype. The chronological sequence of the patient’s clinical manifestations and diagnostic assessments is summarized in Table 1.

### 2.2. Physical Examination

**Figure 1:**
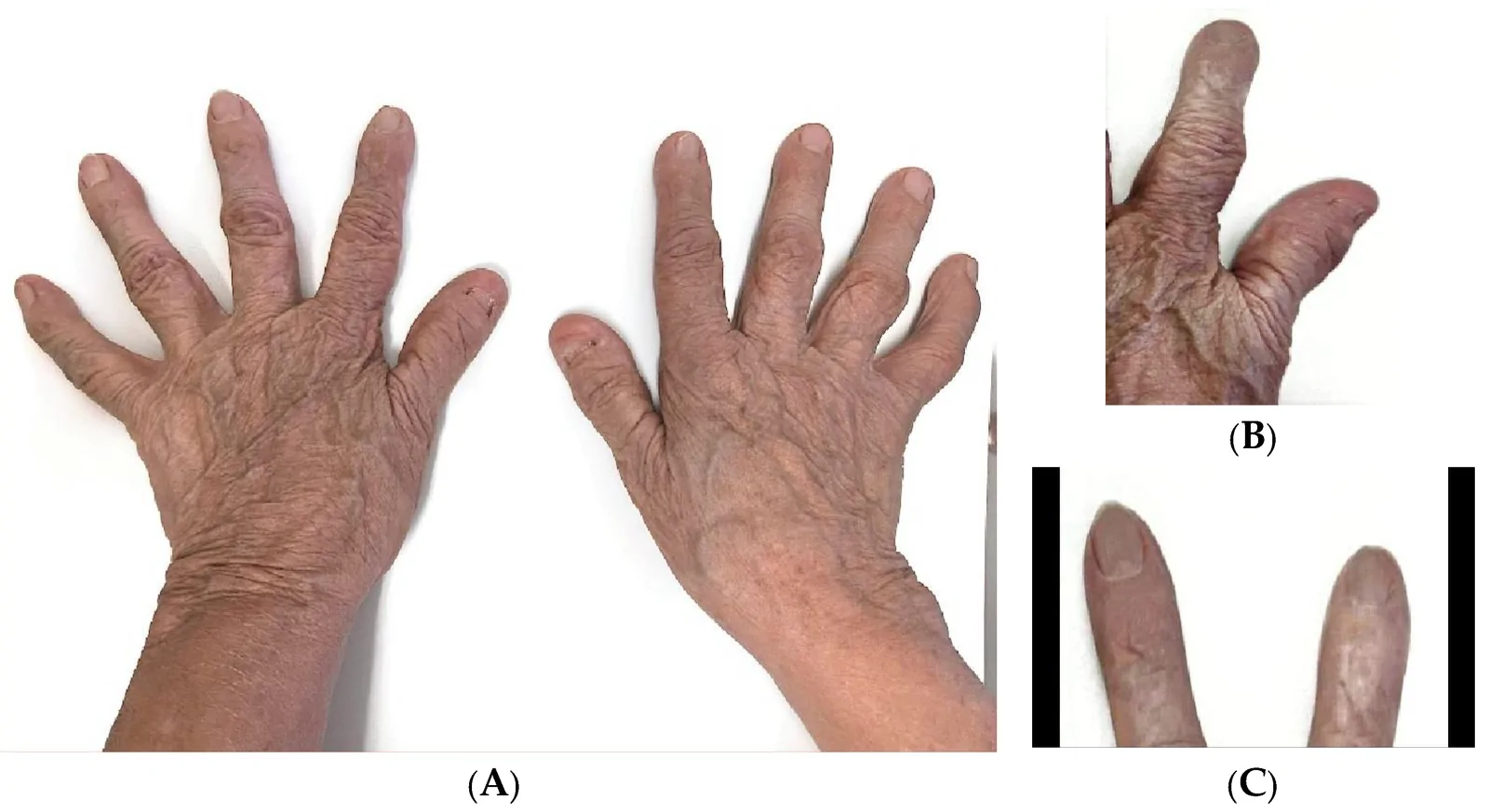
Clinical appearance of the hands in nail-patella syndrome. (**A**) Bilateral nail abnormalities, including hypoplastic thumbnails, bilateral flexion contractures of the fifth fingers, and Heberden nodes. (**B**) Close-up of the left thumb showing marked nail hypoplasia with partial absence of the ulnar portion of the nail plate. (**C**) Close-up of the left second and third fingers showing triangular lunulae, particularly evident on the third fingernail, together with reduced dorsal skin creases over the distal interphalangeal joints.

Examination of the hands revealed bilateral hypoplastic thumbnails with partial absence of the ulnar portion of the nail plates. Triangular lunulae were present, particularly on the third fingernails, together with reduced dorsal skin creases over the distal interphalangeal joints of the second and third fingers. Bilateral flexion contractures of the proximal interphalangeal joints of the fifth fingers were also noted, consistent with camptodactyly. Heberden nodes were present (Figure 1).

Examination of the knees revealed bilateral joint-line tenderness and pain during mobilization. Passive knee flexion was limited to approximately 120° bilaterally. Both patellae were palpable but appeared clinically hypoplastic. Muscle strength was mildly reduced, with quadriceps and hamstrings graded 4+/5 according to the Medical Research Council scale. No instability was detected on varus or valgus stress testing, and Lachman and McMurray tests were negative bilaterally.

Hip examination revealed pain during passive mobilization. Passive hip flexion with the knee flexed was approximately 140° bilaterally, abduction approximately 40°, and internal and external rotation approximately 30°. Mild bilateral weakness of the gluteus medius and iliopsoas muscles was noted, with strength graded 5−/5.

Spinal examination revealed mild dextroconvex lumbar scoliosis. Deep tendon reflexes were normal, and no pathological plantar responses were present.

Elbow extension was limited by approximately 30° on the right and 40° on the left, with additional bilateral restriction of pronation and supination.

Additional findings included bilateral pes planus and superficial hypoesthesia affecting both the hands and feet.

### 2.3. Functional Assessment

Pain intensity during walking was assessed using a 100-mm Visual Analogue Scale (VAS), yielding a baseline score of 70 mm. Knee-related pain, stiffness, and physical function were evaluated using the Western Ontario and McMaster Universities Osteoarthritis Index (WOMAC). The baseline WOMAC total score was 42/96, comprising 7/20 for pain, 4/8 for stiffness, and 31/68 for physical function.

The same assessment instruments were used before and after the 10-day rehabilitation program to evaluate short-term changes in pain and functional status. Outcomes were reported descriptively, without inferential statistical analysis, given the single-patient design.

The baseline findings indicated clinically relevant pain and functional impairment affecting walking and activities of daily living.

### 2.4. Radiographic Assessment

**Figure 2:**
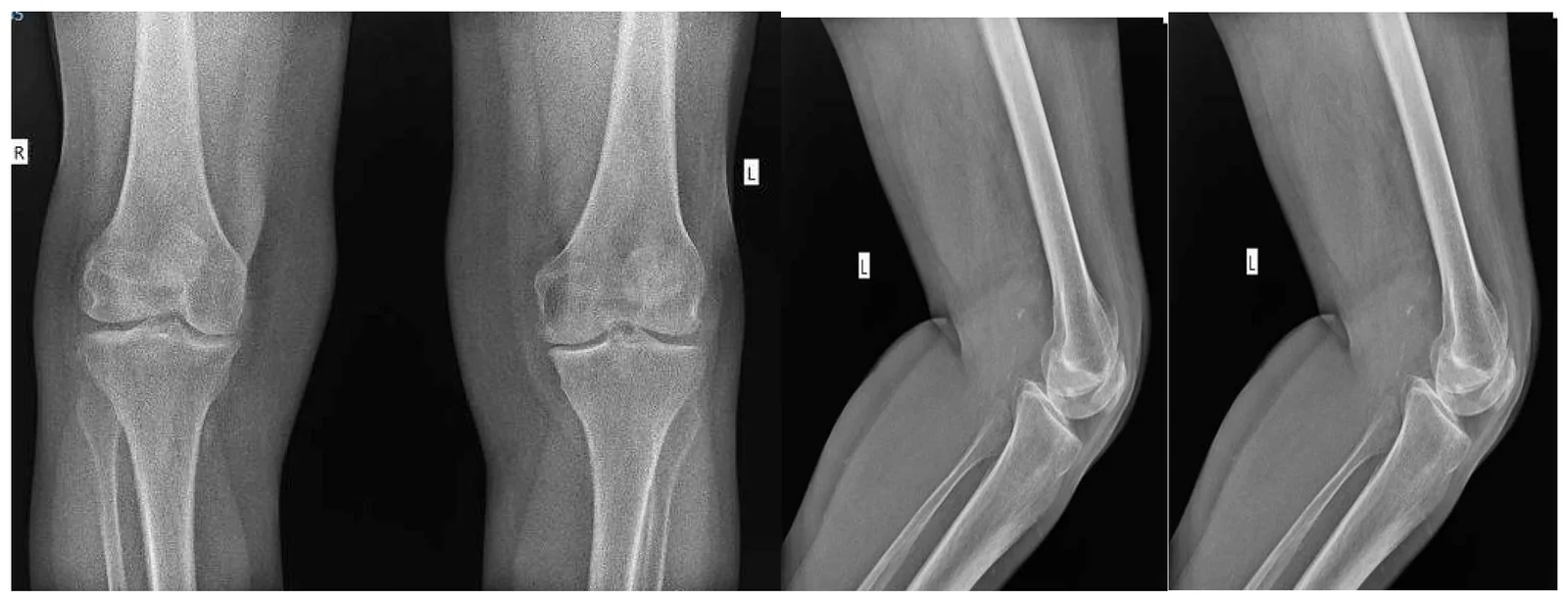
**Bilateral knee radiographs demonstrating patellar abnormalities in nail-patella syndrome.** Bilateral patellar hypoplasia is present, markedly more pronounced on the right, with lateral displacement of the left patella. Associated degenerative changes are consistent with bilateral knee osteoarthritis; R, right; L, left.

Radiographic evaluation of both knees demonstrated bilateral patellar hypoplasia, markedly more pronounced on the right, with lateral displacement of the left patella. Associated bilateral knee osteoarthritis was present and was graded as Kellgren–Lawrence grade 2 on the right and grade 3 on the left (Figure 2).

**Figure 3:**
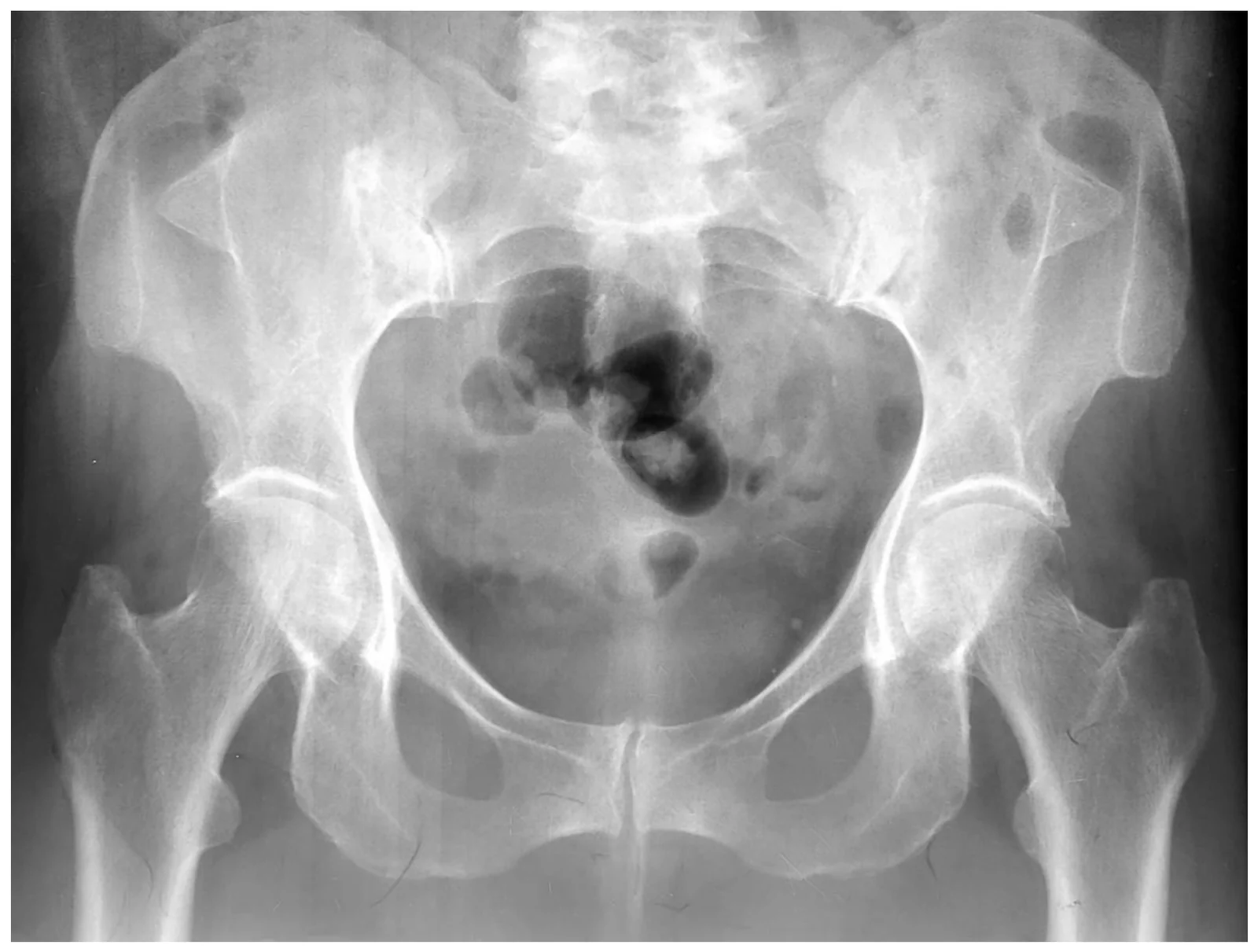
**Bilateral iliac horns in nail-patella syndrome.** Anteroposterior pelvic radiograph showing bilateral, symmetrical, cone-shaped osseous projections arising from the posterolateral aspects of the iliac bones. These characteristic iliac horns represent a highly distinctive radiographic feature of nail-patella syndrome. Mild bilateral degenerative changes in the hip joints are also present.

An anteroposterior pelvic radiograph demonstrated mild bilateral degenerative changes in the hip joints, corresponding to Kellgren–Lawrence grade 2. Importantly, bilateral iliac horns were identified as symmetrical, cone-shaped osseous projections arising from the posterolateral aspects of the iliac bones. This highly characteristic radiographic finding provided a major diagnostic clue supporting nail-patella syndrome (Figure 3).

Radiographic evaluation of the lumbar spine demonstrated mild dextroconvex scoliosis and multilevel degenerative changes, including intervertebral disc space narrowing, marginal vertebral osteophytes, and facet joint osteoarthritis.

Left foot radiographs showed a bifid configuration of the fifth metatarsal in the context of previous surgical correction of postaxial polydactyly, together with post-traumatic deformities of the second and third metatarsals secondary to old healed fractures.

Osteopenia had previously been documented.

### 2.5. Extra-Skeletal Assessment

Bilateral Lester sign was present. Ophthalmological evaluation confirmed bilateral glaucoma, and optical coherence tomography demonstrated bilateral thinning of the retinal nerve fiber layer and ganglion cell layer. The patient had been receiving antiglaucoma treatment for several years.

Neurological manifestations included numbness and tingling affecting the hands and feet. Clinical examination additionally revealed superficial hypoesthesia in the same regions.

Routine laboratory investigations were within normal limits. In particular, urinalysis showed no proteinuria or hematuria, with no documented evidence of renal involvement at the time of evaluation.

### 2.6. Diagnostic Assessment

The diagnosis of nail-patella syndrome was established on clinical and radiographic grounds, based on the coexistence of multiple characteristic findings. These included congenital hypoplastic thumbnails, triangular lunulae, bilateral flexion contractures of the fifth fingers, bilateral patellar hypoplasia, marked limitation of elbow extension, and bilateral iliac horns.

Among these findings, the combination of characteristic nail abnormalities, patellar hypoplasia, elbow involvement, and bilateral iliac horns constituted a highly distinctive diagnostic pattern consistent with NPS. The presence of glaucoma and bilateral Lester sign, together with osteopenia, sensory abnormalities, and a suggestive family history, further supported the multisystem nature of the phenotype.

Molecular genetic testing for a pathogenic variant in *LMX1B* was not performed because, at the time of evaluation, testing was available only through private-sector services and was not publicly reimbursed. The patient declined self-funded testing. Consequently, molecular confirmation was unavailable, and the diagnosis was established on the basis of the characteristic clinical and radiographic phenotype. The diagnostic assessment was based on the integration of clinical findings, radiographic abnormalities, and family history. Although age-related degenerative joint disease could explain some of the patient’s musculoskeletal symptoms, it could not account for the congenital nail abnormalities, patellar hypoplasia, restricted elbow mobility, and bilateral iliac horns. The coexistence of these characteristic features strongly supported the clinical diagnosis of nail-patella syndrome. The absence of molecular confirmation represents a diagnostic limitation. The characteristic clinical, radiographic, and extra-skeletal findings supporting the diagnosis of nail-patella syndrome are summarized in Table 2.

Overall, the coexistence of congenital nail abnormalities, bilateral patellar hypoplasia, elbow involvement, and bilateral iliac horns constituted highly characteristic clinical and radiographic phenotype of nail-patella syndrome.

### 2.7. Therapeutic Intervention and Follow-Up

The patient underwent a 10-day comprehensive medical rehabilitation program consisting of electrotherapy, laser therapy, massage, and therapeutic exercises. The intervention aimed to reduce pain, improve joint mobility, strengthen the lower-limb musculature, correct postural abnormalities, and improve gait. The rehabilitation program was individualized according to the patient’s musculoskeletal limitations, particularly involving the knees, hips, spine, and elbows.

Following rehabilitation, pain intensity during walking decreased from 70 to 50 mm on the 100-mm Visual Analogue Scale (VAS). The total WOMAC score improved from 42/96 to 37/96, with reductions in the pain subscore from 7/20 to 6/20, stiffness subscore from 4/8 to 3/8, and physical function impairment subscore from 31/68 to 28/68.

These findings indicate a short-term improvement in pain and functional status following rehabilitation. However, detailed information regarding the frequency and duration of individual therapeutic procedures was not available in the retrospective documentation. Longer-term follow-up data were also unavailable. Therefore, the sustained effectiveness of the intervention could not be assessed. Rehabilitation remained supportive and symptom-oriented, without modifying the underlying congenital abnormalities associated with nail-patella syndrome.

### 2.8. Patient Perspective

The patient’s personal perspective on the diagnostic process, rehabilitation experience, and perceived treatment outcomes was not formally documented. Consequently, a patient-reported narrative could not be included in this case report.

## 3. Discussion

Nail-patella syndrome (NPS) is characterized by substantial phenotypic variability. In the present case, the diagnosis was supported by the coexistence of congenital thumbnail abnormalities, triangular lunulae, bilateral patellar hypoplasia, restricted elbow mobility, and bilateral iliac horns. The diagnostic value lies in the combination of these characteristic findings rather than in any single manifestation [1,5,6,7,8,9,10].

Among these findings, bilateral iliac horns are particularly informative radiologically and provide a strong diagnostic clue when associated with characteristic nail, patellar, and elbow abnormalities [1,5,6,8,9,10].

The main diagnostic challenge in this patient was the delayed integration of longstanding manifestations into a recognizable multisystem phenotype. Nail and elbow abnormalities had been present since childhood, whereas NPS was first suspected at the age of 43 years following the identification of bilateral iliac horns in one of her grandsons. However, no comprehensive multisystem evaluation or molecular confirmation was performed at that time. This case illustrates how fragmented recognition of characteristic manifestations may delay complete diagnostic assessment, even when several suggestive features are present [1,2,3,4,5].

The musculoskeletal phenotype was clinically relevant, particularly in the context of advanced age and superimposed degenerative joint disease. Radiographic findings were essential for identifying the characteristic skeletal abnormalities of NPS, including bilateral iliac horns and patellar hypoplasia [1,5,6,8,9,10].

Patellar abnormalities constitute a major component of the NPS phenotype. In the present patient, bilateral patellar hypoplasia, more pronounced on the right, and lateral displacement of the left patella were associated with chronic knee pain, restricted flexion, mild muscle weakness, and radiographic osteoarthritis. Altered patellofemoral anatomy may influence joint alignment and load distribution, potentially contributing to degenerative changes [7,12,13,14,15,16,17]. However, a causal relationship between NPS-related skeletal abnormalities and osteoarthritis cannot be established from a single case, particularly given the patient’s advanced age.

The musculoskeletal phenotype was associated with substantial functional impairment. Baseline walking pain was 70 mm on the 100-mm Visual Analogue Scale (VAS), and the WOMAC total score was 42/96. Elbow extension was limited by 30° on the right and 40° on the left, with additional restriction of pronation and supination. These longstanding abnormalities had interfered with the patient’s occupational activities as a tailor, illustrating the cumulative functional burden of the disorder.

The phenotype extended beyond the classical nail–patella–elbow–pelvis pattern. Additional findings included mild lumbar scoliosis, multilevel degenerative lumbar changes, bilateral pes planus, a bifid configuration of the left fifth metatarsal following surgical correction of postaxial polydactyly, osteopenia, and sensory abnormalities. Although these manifestations are not individually diagnostic, their coexistence illustrates the broad clinical spectrum encountered in NPS.

Ophthalmological involvement was particularly relevant. The patient had bilateral glaucoma, bilateral Lester sign, and thinning of the retinal nerve fiber and ganglion cell layers documented by optical coherence tomography. These findings emphasize the importance of continued ophthalmological surveillance in patients with NPS, particularly given the recognized association with ocular hypertension and glaucoma [1,5,18,19].

Renal involvement represents another clinically important manifestation of NPS, ranging from isolated proteinuria or hematuria to progressive nephropathy in a subset of affected individuals [1,4,6,20,21,22,23]. In the present patient, routine urinalysis showed no proteinuria or hematuria. However, a urinary albumin-to-creatinine ratio measurement was unavailable, limiting the sensitivity of the renal assessment. The absence of detectable renal abnormalities on routine urinalysis does not exclude renal involvement or the diagnosis of NPS and is consistent with the variable expression of the disorder [1,2,3,4,5].

Identification of a pathogenic *LMX1B* variant may provide molecular confirmation of NPS, particularly in atypical presentations and during family screening [1,2,3,20,21,24,25,26,27,28,29]. In this case, genetic testing was available only through private-sector services and was not publicly reimbursed at the time of evaluation. The patient declined self-funded testing, precluding molecular confirmation and genotype–phenotype correlation. Nevertheless, the characteristic combination of nail abnormalities, patellar hypoplasia, elbow involvement, and bilateral iliac horns strongly supported the clinical and radiographic diagnosis.

Family history provided an additional diagnostic clue. Bilateral iliac horns had reportedly been identified radiographically in one grandson, raising the possibility of familial involvement. However, other relatives had not undergone systematic clinical or genetic evaluation, and a formal pedigree was unavailable. Family assessment and genetic counseling would therefore be appropriate to identify potentially affected relatives and evaluate intrafamilial phenotypic variability [1,2,3,20,27].

The particular value of this case lies in the prolonged interval between congenital manifestations and comprehensive multisystem characterization. Clinical, radiographic, ophthalmological, neurological, functional, and familial findings converged to support a coherent diagnosis in an older adult. This highlights the importance of reconsidering longstanding congenital abnormalities collectively when additional organ-system manifestations emerge.

From a rehabilitation perspective, the combination of chronic pain, restricted joint mobility, muscle weakness, and gait impairment supports individualized functional management. Following a 10-day rehabilitation program, walking pain decreased from 70 to 50 mm on the VAS, while the WOMAC total score improved from 42/96 to 37/96. These findings indicate short-term symptomatic and functional improvement but do not establish sustained treatment effectiveness or modification of the underlying congenital disorder.

More broadly, this case illustrates how multisystem disorders may remain incompletely recognized when apparently unrelated manifestations are assessed in isolation. Integrating findings across specialties is essential for comprehensive diagnosis and management, a principle relevant to other complex multisystem conditions [30].

### 3.1. Clinical Implications of Early Recognition and Rehabilitation Strategy

Early recognition of NPS has implications beyond establishing the diagnosis. Although congenital skeletal abnormalities cannot be reversed, timely identification may facilitate surveillance for renal and ophthalmological complications, assessment of musculoskeletal abnormalities, and implementation of individualized rehabilitation measures. Recognition of the hereditary nature of the disorder may also prompt family evaluation and genetic counseling.

Rehabilitation in NPS should address the multisite musculoskeletal phenotype rather than focus exclusively on isolated knee symptoms. Patellar hypoplasia or maltracking, restricted elbow mobility, altered lower-limb alignment, spinal abnormalities, foot deformities, muscle weakness, and gait disturbances should be considered when developing individualized management strategies [1,13,14,31]. Treatment goals should emphasize pain reduction, preservation of functional joint mobility, muscle strengthening, stabilization, and prevention of secondary mechanical overload.

Joint protection and ergonomic adaptation are particularly relevant in patients with longstanding skeletal abnormalities. Avoidance of repetitive mechanical overload and modification of activities that aggravate symptoms may help reduce functional limitations. In patients with restricted elbow mobility, adaptations of occupational and daily activities may be preferable to forceful attempts to restore normal joint motion.

A kinetic-chain approach may also be beneficial because abnormalities involving the hips, knees, feet, and spine can interact and influence load distribution and gait. Rehabilitation strategies may therefore include strengthening of the hip abductors and extensors, quadriceps training, postural correction, appropriate foot support, and gait re-education, according to individual clinical findings.

In the present patient, the rehabilitation program included electrotherapy, laser therapy, massage, and therapeutic exercises, with an emphasis on pain control, joint mobility, muscle strengthening, postural correction, and gait improvement. Following the 10-day intervention, VAS and WOMAC scores demonstrated short-term improvement. However, detailed treatment parameters, including the frequency and duration of individual procedures, were unavailable in the retrospective documentation. Consequently, the intervention cannot be reproduced precisely, and its longer-term effectiveness remains uncertain.

These findings support an individualized, multidisciplinary approach to functional management in NPS while highlighting the need for detailed treatment documentation and longitudinal evaluation.

### 3.2. Limitations

This case report has several limitations. First, the single-patient design precludes causal conclusions regarding the relationship between congenital skeletal abnormalities associated with NPS and the observed degenerative joint changes. Second, molecular genetic testing for *LMX1B* was not performed because the patient declined self-funded testing, preventing molecular confirmation and genotype–phenotype correlation.

Third, detailed longitudinal renal and ophthalmological data were unavailable, and renal assessment was limited to routine urinalysis without urinary albumin-to-creatinine ratio measurement. Furthermore, although short-term improvements in VAS and WOMAC scores were documented following rehabilitation, detailed treatment parameters and long-term follow-up data were unavailable, limiting assessment of treatment reproducibility and sustained effectiveness.

Finally, potentially affected family members were not systematically evaluated or genetically tested, and the patient’s personal perspective was not formally documented.

Despite these limitations, the case highlights the diagnostic importance of integrating characteristic clinical and radiographic findings, particularly bilateral iliac horns, in older patients with longstanding multisystem manifestations.

## 4. Conclusions

Nail-patella syndrome is a rare hereditary multisystem disorder that may remain incompletely recognized for decades, particularly when congenital abnormalities are evaluated separately or obscured by age-related degenerative changes. In the present case, the coexistence of characteristic nail abnormalities, bilateral patellar hypoplasia, restricted elbow mobility, and bilateral iliac horns provided a highly distinctive clinical and radiographic pattern supporting the diagnosis.

Associated ophthalmological, neurological, and musculoskeletal manifestations further highlighted the importance of comprehensive multisystem assessment. Recognition of these characteristic findings should prompt appropriate renal and ophthalmological surveillance, evaluation of musculoskeletal complications, and consideration of genetic counseling and family assessment.

Although individualized rehabilitation was associated with short-term improvements in pain and functional outcomes, long-term effectiveness could not be determined. This case emphasizes the importance of early recognition, multidisciplinary management, and continued surveillance in patients with nail-patella syndrome.

## Figures and Tables

**Table 1:** Timeline of the Patient’s Clinical History.

Time Point	Clinical Event
Childhood	Congenital thumbnail hypoplasia and long-standing restriction of elbow extension
Age 43 years	Nail-patella syndrome first suspected after bilateral iliac horns were identified in one grandson, prompting reassessment of the patient’s longstanding nail and musculoskeletal abnormalities as part of a possible hereditary multisystem disorder
In her fifties	Diagnosis of glaucoma; bilateral Lester sign documented on ophthalmological evaluation
Approximately 20–25 years before the current evaluation	Onset of bilateral mechanical knee pain, with progressive worsening
Approximately 10 years before the current evaluation	Development of mechanical pain and functional limitation involving the lumbosacral spine, hips, and ankles, accompanied by increasing difficulty with walking
Current evaluation at age 76 years	Comprehensive clinical, functional, radiographic, ophthalmological, neurological, and multisystem assessment confirming a highly characteristic NPS phenotype

**Table 2:** Diagnostic Features of Nail-Patella Syndrome Identified in the Present Patient.

Domain	Findings in the Present Patient	Diagnostic Relevance
Nail phenotype	Bilateral hypoplastic thumbnails; partial absence of the ulnar portion of the nail plates; triangular lunulae, particularly on the third fingernails	Characteristic nail phenotype of NPS
Hand findings	Reduced dorsal creases over the distal interphalangeal joints of the second and third fingers; bilateral flexion contractures of the fifth fingers	Additional clinical features supporting the syndromic phenotype
Patellar phenotype	Bilateral patellar hypoplasia, markedly more pronounced on the right; lateral displacement of the left patella	Major skeletal manifestation of NPS
Elbow phenotype	Extension deficit of 30° on the right and 40° on the left; restricted pronation and supination bilaterally	Characteristic musculoskeletal manifestation of NPS
Pelvic radiographic findings	Bilateral symmetrical iliac horns	Pathognomonic radiographic feature strongly supporting NPS
Knee osteoarthritis	Kellgren–Lawrence grade 2 on the right and grade 3 on the left	Associated degenerative musculoskeletal finding
Hip and spine findings	Bilateral hip osteoarthritis, Kellgren–Lawrence grade 2; mild dextroconvex lumbar scoliosis; multilevel degenerative lumbar changes	Additional musculoskeletal involvement
Foot findings	Bilateral pes planus; bifid configuration of the left fifth metatarsal; residual lateral forefoot deformity following previous correction of postaxial polydactyly	Additional skeletal abnormalities within the overall phenotype
Ophthalmological findings	Bilateral glaucoma; bilateral Lester sign; bilateral thinning of the retinal nerve fiber layer and ganglion cell layer on optical coherence tomography	Extra-skeletal involvement supporting the multisystem phenotype
Neurological/sensory findings	Paresthesia and superficial hypoesthesia of the hands and feet	Additional extra-skeletal manifestations
Bone status	Osteopenia	Associated skeletal finding
Renal assessment	Normal routine urinalysis; no proteinuria or hematuria	No clinically evident renal involvement at the time of evaluation
Functional impairment	Walking pain VAS: 70 mm on a 100-mm scale; WOMAC total score: 42/96	Demonstrates the functional burden of the musculoskeletal phenotype
Family history	Bilateral iliac horns identified radiographically in one grandson	Supports possible familial involvement
Molecular testing	*LMX1B* genetic testing not performed	Molecular confirmation unavailable; diagnosis based on clinical and radiographic findings

## Data Availability

The data supporting the findings of this case report are available from the corresponding author upon reasonable request, subject to applicable privacy and ethical restrictions.

## Supplementary Materials

The following supporting information can be downloaded at: https://www.mdpi.com/article/10.3390/jcm15207861/s1, Table S1: CARE 2013 reporting checklist [11].
