# Supplementary material for: Nail-Patella Syndrome in a 76-Year-Old Woman: Multisystem Phenotype, Diagnostic Delay, and Clinical Implications
Source: J Clin Med. 2026 Oct 12;15(20):7861. doi: 10.3390/jcm15207861 (PMC13705258; doi:10.3390/jcm15207861)
Supplement: Supplementary file 1 [file jcm-15-07861-s001.zip › jcm-4573446-supplementary.pdf]

Table S1. CARE 2013 reporting checklist — completed

Manuscript: Nail-Patella Syndrome in a 76-Year-Old Woman: Multisystem Phenotype, Diagnostic Delay, and Clinical Implications—A Case Report

Manuscript identifier: jcm-4573446 | Basis: author-supplied final(1).docx (16 rendered pages)

This reporting map follows the 2013 CARE checklist. "Not documented" means that information was not present in the manuscript; it must not be inferred as a negative clinical finding.

CARE item	Reporting element	Manuscript location	Status / explanation
**1**	Title identifies the condition and includes "case report"	Title, p. 1	Reported
**2**	Two to five relevant keywords, including "case report"	Keywords, p. 2	Reported — five keywords, including "case report"
**3a**	Abstract states what makes the case noteworthy	Abstract, p. 2	Reported
**3b**	Abstract summarizes main symptoms and clinical findings	Abstract, p. 2	Reported
**3c**	Abstract summarizes diagnosis, intervention and outcomes	Abstract, p. 2	Reported; 10-day rehabilitation, VAS and WOMAC
**3d**	Abstract provides the principal clinical lesson	Abstract, p. 2	Reported
**4**	Introduction explains the case relevance with literature	Section 1, p. 3	Reported
**5a**	Non-identifying demographic and patient-specific details	Section 2.1, p. 4	Reported; 76-year-old woman
**5b**	Presenting symptoms and concerns	Section 2.1, p. 4	Reported
**5c**	Relevant medical, family and psychosocial history	Section 2.1 and Table 1, pp. 4–5	Partial — medical/family/occupation given; psychosocial detail limited
**5d**	Previous relevant interventions and outcomes	Section 2.1, pp. 4–5	Partial — prior foot surgery and glaucoma treatment noted; outcomes only partly recorded
**6**	Relevant examination and clinical findings	Sections 2.2 and 2.5, pp. 5–7	Reported
**7**	Timeline of history and care events	Table 1, p. 4	Reported
**8a**	Diagnostic methods (examination, imaging, laboratory, functional scales)	Sections 2.2–2.6, pp. 5–8	Reported
**8b**	Diagnostic difficulties, including access barriers	Sections 2.1 and 2.6, pp. 4 and 8	Reported — delayed recognition and self-funded genetic testing
**8c**	Diagnostic reasoning and alternatives	Section 2.6, p. 8	Reported — age-related degenerative disease discussed; no systematic differential-workup claimed
**8d**	Prognostic or staging features, where applicable	Sections 2.4–2.6; Table 2, pp. 6–9	N/A for formal staging; joint OA grades and other findings are reported
**9a**	Types of interventions administered	Section 2.7, p. 9	Reported — electrotherapy, laser, massage, exercises
**9b**	Administration details (duration, frequency, intensity)	Section 2.7, p. 9	Partial — overall 10 days; detailed modality parameters not available
**9c**	Changes to treatment and	Section 2.7, p. 9	Not documented

	reasons		
**10a**	Clinician- and patient-assessed outcomes	Sections 2.3 and 2.7, pp. 6 and 9	Partial — VAS/WOMAC pre/post available; narrative patient perspective absent
**10b**	Important follow-up test results	Section 2.7, p. 9	Partial — end-of-program scales; no longer-term tests
**10c**	Adherence and tolerability	Section 2.7, p. 9	Not documented
**10d**	Adverse or unexpected events	Section 2.7, p. 9	Not documented; cannot assert none occurred
**11a**	Strengths and limitations of the case	Section 3.2, p. 13	Reported
**11b**	Relevant medical literature and comparison	Section 3, pp. 10–13	Reported
**11c**	Reasoning behind conclusions; alternative explanations	Sections 3 and 3.2, pp. 10–13	Reported
**11d**	Key lesson(s)	Sections 3 and 4, pp. 10–14	Reported
**12**	Patient perspective in the patient's own account	Section 2.8, p. 9	Not available; absence explicitly stated
**13**	Informed consent for publication	Informed Consent Statement, p. 14	Reported in manuscript; confirm signed form available if journal asks

Items to review before submission

- Keywords (item 2): five keywords are now provided, including "case report"; this item is fulfilled.
- Intervention details (9b), treatment changes (9c), adherence/tolerability (10c), and adverse events (10d) cannot be completed beyond the existing records. Do not report "none" unless documented.
- Patient perspective (12) was not documented; the manuscript explicitly discloses this.
- Page locations are indicative and may shift if final formatting changes. Section names remain reliable.

Reference: Gagnier JJ et al. The CARE Guidelines: Consensus-based Clinical Case Reporting Guideline Development. BMJ Case Rep. 2013. DOI: 10.1136/bcr-2013-201554.
